# Anaemia: A Useful Indicator of Neglected Disease Burden and Control

**DOI:** 10.1371/journal.pmed.0040231

**Published:** 2007-08-14

**Authors:** Imelda Bates, Stephen McKew, Faruk Sarkinfada

## Abstract

The authors argue that one way of evaluating the effectiveness of an intervention aimed at controlling neglected tropical diseases would be to assess its impact on anemia prevalence.

The world's neglected tropical diseases (NTDs; Table 1) are becoming less neglected. Many millions of dollars and drugs are now committed to their control, and an open-access journal dedicated to NTDs, *PLoS Neglected Tropical Diseases*, has been launched (http://www.plosntds.org/). NTDs are characterised by their prevalence in rural areas of low-income countries and fragile states, their tendency to promote poverty, and the absence of commercial markets for tools that would control them, even though such tools exist and their success has been demonstrated [1].

**Table 1:**
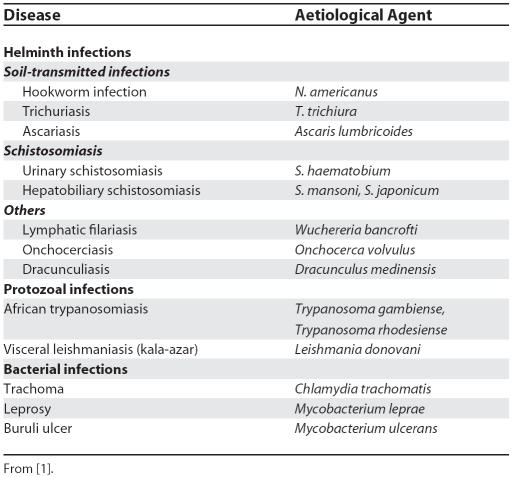
Neglected Tropical Diseases in Africa and Their Major Aetiological Agents

NTD programmes are effective development investments because interventions can be delivered for less than $US1 per person per year and economic rates of return are 15%–30% [2]. In addition, individual NTD programmes are often able to share activities such as drug distribution because of geographical overlap between their target populations [1,3,4]. These benefits were cited in the World Health Assembly resolution WHA 54.19, which recommends the provision of essential drugs against schistosomiasis and soil-transmitted helminthiasis for 100% of school children at risk by 2010 [5].

NTD programmes need to be able to demonstrate that they have met their objectives [6]. In general, each programme has established its own monitoring and evaluation system, such as microscopic counting of eggs in urine or faeces for schistosomiasis or hookworm, night blood films for microfilaria, or skin snips for onchocerciasis. Apart from the difficulties of obtaining samples and transporting them to a laboratory or setting up microscopy services within communities, the high day-to-day variability of biological measurements within individuals makes such methods of monitoring tedious and insufficient on their own. These indicators evaluate infection rather than morbidity [6], and David Molyneux and colleagues have suggested that well-being and equity would be more appropriate indicators to evaluate changes in public health [1].

Prevalence of anaemia could be added to this choice of indicators because: (1) such prevalence is objective and quantifiable; (2) anaemia is a major complication of several NTDs; (3) it can be measured even in the most remote areas; and (4) it changes in a predictable fashion with alterations in disease burden. Because the prevalence of anaemia increases with worsening socio-economic status [7], it can be used to assess whether an intervention has reached the poorest communities in areas where NTDs are a major cause of anaemia.

## Usefulness of Anaemia as an Indicator for NTDs

In developing countries, over half of all children and pregnant women are anaemic [8]. Even mild anaemia negatively affects health, productivity, development, and immune function, and it is particularly detrimental to children, pregnant women, and individuals with HIV infection [9]. The aetiology of anaemia is multifactorial in developing countries and includes deficiencies of micronutrients (e.g., iron, folate, vitamin B12); haemoglobinopathies; infections and chronic diseases (e.g., malaria, NTDs, HIV, tuberculosis); and cancer. Mean haemoglobin levels and/or the prevalence of anaemia will only be useful indicators for NTD programmes if they reflect changes in the burden of NTDs in the target populations in response to specific interventions.

## Relationship between NTDs and Anaemia

Many NTDs cause anaemia either directly through blood loss or indirectly through bone marrow suppression, inflammation, hypersplenism, haemolysis, or anorexia [8,10]. Anaemia is particularly common in individuals infected with soil-transmitted helminths or schistosoma. The amount of blood loss in hookworm infections (Necator americanus and Ancylostoma duodenale) is strongly and linearly correlated with worm load and faecal egg count, and even light infections contribute significantly to anaemia [11]. Trichuris trichiura also causes intestinal blood loss, and schoolchildren with heavy T. trichiura infections have a higher prevalence of anaemia (haemoglobin [Hb] <11 g/dl) than uninfected controls [12].

Where transmission in communities is high, NTDs are a major cause of mild and moderate anaemia (i.e., Hb 7–11.9 g/dl). For example, 66% of pregnant Tanzanian women [13] and 50%–62% of Tanzanian children [14,15] living in high-risk areas for helminth and/or schistosomiasis infections have anaemia. Schistosoma mansoni, S. japonicum, and S. haematobium cause intestinal or urinary blood loss. Studies from Kenya, Niger, Tanzania, and elsewhere have shown that, despite the presence of confounding factors, haemoglobin levels of children and pregnant women correlate negatively with egg count in both S. mansoni and S. haematobium [9,14]. Infection with several parasites at once (“polyparasitism”) is common in tropical countries, particularly in poor communities. There is an inverse relationship between the number of parasites in an infected person and their Hb level. For example, low-intensity polyparasite infections in children in the Philippines are associated with 5-fold increases in prevalence of anaemia (Hb <11 g/dl) [16]. Moderate/high intensity infections with three or four soil-transmitted helminths and S. japonicum are associated with 8-fold increases in anaemia.

In addition to its use in individual NTD programmes, anaemia monitoring could also be a useful tool when NTD control is integrated with other major diseases. Anaemia is a major cause of morbidity in co-infections with NTDs and malaria, HIV, and/or tuberculosis [2]. Up to 35% of anaemia cases in African children are due to malaria [17]. Anaemia is an independent prognostic marker in HIV infection; it also contributes to progression of both HIV and tuberculosis [18], and in some cases reversal of anaemia can reduce the risk of premature death from complications of HIV [18,19].

## Effects of Programmes to Control NTDs on Anaemia Prevalence

Providing chemotherapy for groups at high risk of soil-transmitted helminths improves growth and iron stores in children and reduces anaemia in pregnant women [20]. The impact of treatment on anaemia is well established for hookworm infections but seems to be less marked for trichuriasis, particularly outside Africa [21–23]. The effect of treatment for schistosomiasis on haemoglobin levels and prevalence of anaemia is difficult to disentangle from the confounding effects of other conditions causing anaemia, but overall schistosomiasis treatment alone appears to be beneficial for reducing the prevalence of anaemia [8].

Reduction in the prevalence of anaemia is used as a measurable target in programmes to reduce morbidity due to schistosomiasis and helminthiasis (see examples in Table 2 and relationship between NTDs and anaemia in Table 3) [4,6,24]. Such a target is used because it reflects the aim of the programmes to reduce disease morbidity rather than transmission [4,20]. In integrated programmes that used control measures for more than one NTD, a combination of albendazole and praziquantel significantly reduced anaemia (defined as Hb <12 g/dl) in Tanzanian children living in an area of high endemicity for helminth and schistosomiasis infections [25]. In Uganda, the same drug combination produced a 52% reduction in the proportion of cases of anaemia; the most marked improvements occurred among the most heavily infected children [26].

**Table 2:**
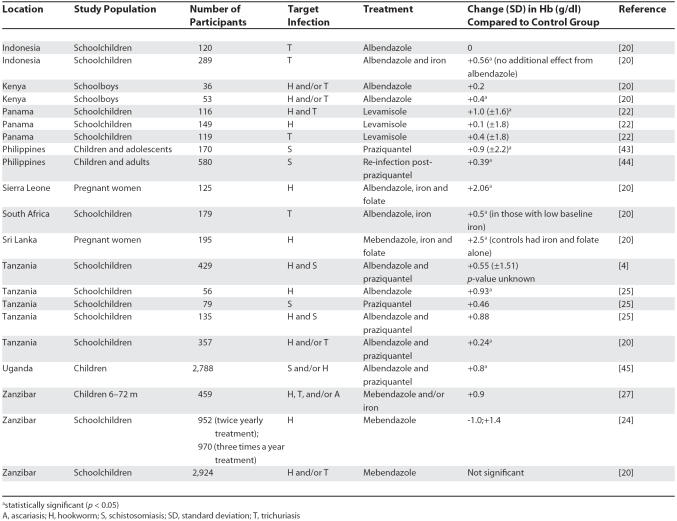
Illustrative Examples of Studies That Have Investigated the Effects of NTD Interventions on Anaemia

**Table 3:**
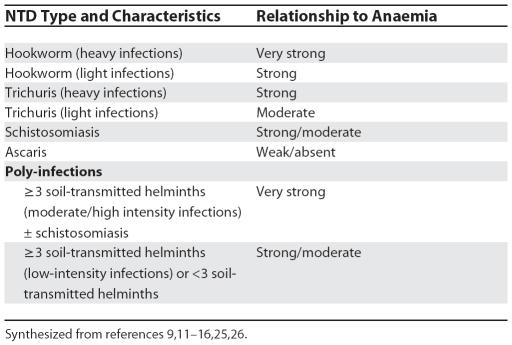
Summary of the Relationship between NTDs and Anaemia

Differences in study design, monitoring indicators, and ancillary treatments such as iron supplements have contributed to conflicting results in assessments of the outcomes of NTD programmes. Many of the populations living where NTDs are prevalent are iron deficient, so treatment which aims solely to reduce the burden of infection may slow the rate of iron loss but will not increase red cell production [20]. Published studies have suggested that adding iron supplements to deworming treatment may improve haemoglobin levels. In Sierra Leone, the addition of iron to anthelminthic treatment significantly reduced the fall in haemoglobin in pregnant women compared to anthelminthic treatment alone [27].

The impact of combining NTD treatment with iron supplementation is not always predictable. In children aged under two years in Zanzibar, iron supplements alone did not reduce anaemia, probably because of associated factors such as inflammation and malaria, whereas mebendazole alone induced a significant reduction in anaemia even in children with light helminth infections [28]. This finding was thought to be related to the high risk of this age group for wasting malnutrition and for acquiring first time-helminth infections, which may induce proinflammatory mediators. In schoolchildren living in areas where hookworm infections are endemic, 25%–73% of occurrences of mild to severe anaemia and 35% of occurrences of iron deficiency are due to hookworm infection. Overall evidence is accumulating that iron supplementation should be included with anthelminthic therapy in NTD treatment programmes [14]. However, given that such supplementation may be associated with increased severity of infectious disease in the presence of malaria and/or undernutrition in certain sub-groups [29], the benefits need to be optimised without incurring additional risks.

**Box 1. NTD Programme Requirements for a Method to Measure Haemoglobin Concentrations**

A test that is to be used by NTD programmes for large-scale monitoring and rapid scale-up should:

- be accurate enough to detect the anticipated changes in haemoglobin levels induced by interventions for NTDs
- not require mains electricity
- be able to be performed with minimal training and supervision
- use whole blood so that no dilution steps are required

## Measuring Anaemia in Resource-Poor Settings

Because NTDs often co-occur with iron deficiency, treatment that is not combined with iron supplementation is likely to reduce, but not reverse, the associated fall in haemoglobin levels. The changes in haemoglobin level induced by treatment that aims only to reduce NTD infection may therefore not be very dramatic (see Table 2), so the method chosen for large-scale screening must be able to detect subtle changes in haemoglobin levels (Box 1).

The Haemoglobin Colour Scale (HCS) has been designed for field situations in resource-poor countries. The colour of a finger prick blood sample, soaked into special chromatography paper, is compared with high-quality digital reproductions of blood of known haemoglobin concentration soaked into the same type of paper (Figure 1). The colours of the haemoglobin samples are represented in 2 g/dl increments from 4 g/dl to 14 g/dl. The HCS can be used by non-laboratory health workers after only a few hours training and is durable in dusty, hot, dry, and humid conditions; recurrent costs are less than $0.05 a test [30]. A systematic review suggests that the HCS is better than clinical diagnosis for detecting mild and moderate degrees of anaemia in children and pregnant women [31,32] and avoids the need to collect and transport venous samples [33]. Drawbacks of the HCS are that it requires specific chromatography paper and good natural light. The HCS is too imprecise to detect incremental changes in haemoglobin less than 1 g/dl, and so its effectiveness within NTD programmes would need to be assessed in field trials.

**Figure 1:**
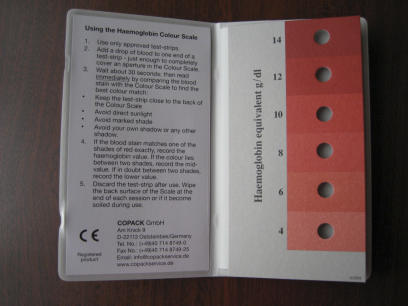
Haemoglobin Colour Scale The colour of a finger prick blood sample, soaked into chromatography paper, is compared with the colour of blood of known haemoglobin content depicted on the scale in 2 g/dl increments from 4 g/dl to 14 g/dl.

The haemoglobin method that has been most widely used in field situations in NTD programmes (e.g., [4,13–15]) is the HemoCue system. This is a small battery-operated machine which produces a direct read-out of haemoglobin to one decimal place in a few seconds, using a drop of blood in a plastic cuvette. The HemoCue method is robust, accurate (correlation coefficient 0.995 in comparison to international reference method), and has an in-built quality checking mechanism. In routine practice and large field surveys it gives consistent results [34], and is very user-friendly. The new HemoCue Hb 301 system (Figure 2), which has been developed specifically for use in tropical countries, operates in temperatures up to 50 °C, in dusty and humid conditions, and each test costs about $US0.50. Cuvettes are hygroscopic and need to be stored in their airtight containers.

**Figure 2:**
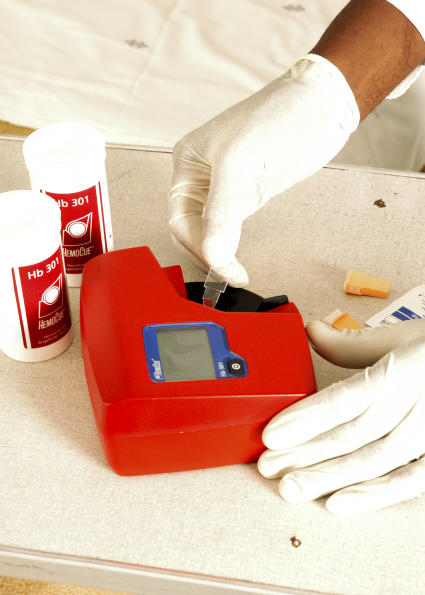
HemoCue Hb 301 A drop of whole blood is introduced into a plastic cuvette by capillary action. The cuvette is placed in the machine, which produces a direct read-out of haemoglobin to one decimal place. (Photo: HemoCue)

The direct cyanmethaemoglobin method is the gold standard for haemoglobin measurement [35], but its use in surveys in remote areas is limited because it requires accurate dilution of the blood sample and electrical power for the spectrophotometer. In practice these constraints may mean that venous samples have to be collected, transported to a laboratory, and analysed retrospectively within a few hours [34]. To overcome these difficulties, an indirect method has been developed in which the blood is dried on filter paper for transportation and then re-dissolved in the laboratory for measurement. But this indirect method significantly overestimates the prevalence of anaemia, probably because some blood remains on the filter paper after the dissolving process [34]. If the method is used for studies of anaemia prevalence, it can produce misleading results [36], and is therefore not suitable as an NTD monitoring tool.

Clinical diagnosis, the Tallqvist method, the copper sulphate method, and the Lovibond (undiluted) comparator method are alternative techniques for measuring haemoglobin that do not require a dilution step, have been evaluated in primary care settings, and meet many of the specifications outlined in Box 1 [37,38]. Clinical diagnosis of anaemia by assessment of the colour of the conjunctiva, nail beds, or palms is insufficiently sensitive or specific to be used for monitoring changes in prevalence of mild and moderate anaemia [39]. Of the other techniques, only the Lovibond undiluted comparator may have satisfactory precision and accuracy [38], but it requires an exact volume of 0.03 ml of whole blood, which is difficult to achieve in field situations.

## Conclusions: Next Steps for Policy and Research

Anaemia is the second most common cause of disability in the world [40], and is reflected in several of the global Millennium Development Goals [41]. The association between anaemia and NTD burden and control is well established for helminthiasis and schistosomiasis, and there are examples of these programmes using population changes in haemoglobin levels as a monitoring tool.

But iron deficiency often accompanies NTDs, so unless these programmes incorporate iron supplementation into their activities they will only be able to achieve a reduction in the rate of decrease of haemoglobin. Therefore the relationship between anaemia and the burden of other NTDs needs to be more clearly established before these programmes can reliably use anaemia as a monitoring tool [42].

Although anaemia could be a valuable component of an NTD monitoring toolkit, it should probably not be used as the sole indicator because confounding factors affecting the aetiology of anaemia, particularly dietary iron deficiency, will reduce its sensitivity and specificity. Drug distribution and compliance is therefore likely to remain the major tool for programme monitoring. Because anaemia is included in demographic health surveys, and is such an important health indicator in programmes for addressing major nutrition, malaria, and HIV, haemoglobin monitoring could be valuable in evaluating other disease control programmes.

Several questions must be answered before anaemia can be firmly recommended as a monitoring tool for NTD programmes. Existing models of NTDs are generally considered to be cost-effective because they use minimal resources, but any additional activity such as haemoglobin measurement risks over-burdening the system. Anaemia as a monitoring tool therefore needs to be carefully evaluated in pilot projects and its cost-effectiveness determined before planning for scale-up. The impact of NTD programmes on anaemia may be enhanced by careful incorporation of iron supplementation, but this has resource implications for programmes that are currently cost-effective. Data on cost-effectiveness are available for the drug delivery components of integrated NTD programmes, but not for other evaluation components. Further studies are needed to examine the implications of incorporating haemoglobin measurements and possibly also iron supplementation into existing programmes.

The HemoCue system is by far the most widely used and accepted haemoglobin technology for NTD programme evaluation and is able to accurately reflect small changes in haemoglobin concentration. The new HemoCue Hb 301 tropical system is also less expensive than the older Hb 201 system. The HCS is simple and inexpensive, but it is not sufficiently sensitive to small changes in haemoglobin to be used on its own. A combination of the two systems, using the HCS for mass screening and the HemoCue Hb 301 for accurate measurements and quality control, has yet to be evaluated but may be a reasonable approach. Rigorous standardisation of both methods is essential and can be achieved through training and regular supervision of staff.

There will inevitably be local variations in the prevalence of NTDs and anaemia, as well as in the availability of resources and technical skills to deliver and monitor NTD programmes. Any mechanisms for assessing the prevalence of anaemia should therefore be evaluated in real-life situations against pre-determined criteria that are derived from the overall objectives of the NTD programmes. Such evaluation is particularly important because it cannot be assumed that costs and effectiveness will remain constant as NTD programmes are scaled up and access more remote areas [26].

## Abbreviations

Hb: haemoglobin
HCS: Haemoglobin Colour Scale
NTD: neglected tropical disease
